# Investigation of the insulation performance of lightweight concrete with silica aerogel additives produced using volcanic tuff waste

**DOI:** 10.1038/s41598-026-51748-9

**Published:** 2026-06-03

**Authors:** Omer Can, Selçuk Çimen

**Affiliations:** Vocational school of technical sciences, Department ofconstruction, Çanakkale onsekiz mart university, Çanakkale, Türkiye

**Keywords:** Lightweight concrete, Bayburt stone (tuffite), Silica aerogel, Insulation properties, Thermal conductivity, Sustainable construction, Energy science and technology, Engineering, Environmental sciences, Materials science

## Abstract

This paper examined how the addition of silica aerogel would impact the performance of lightweight concrete made using Bayburt stone (volcanic tuff) aggregate in insulation. A silica-based gel material was produced by the synthesis of Bayburt stone by the sol-gel process and then added to lightweight concrete mixtures prepared by Taguchi L8 experimental matrix. Signal-to-noise ratios and ANOVA were used to determine factors’ contribution within the chosen design space by analysing the effects of aerogel ratio, cement dosage and water to cement ratio, on unit weight, thermal conductivity, ultrasonic pulse velocity and compressive strength. The findings revealed that the higher the content of aerogel, the lower the unit weight and thermal conductivity by up to 46 per cent at the expense of the reference mixture, which signified better thermal insulation. The porosity percentage of the tested mixtures rose to 31.48%, and compressive strength dropped with the increase in the content of aerogel. Thermogravimetric data indicated that mixtures with aerogel experienced lower total mass loss, but these data are not taken as evidence of long-term stability at all, but only as the differences in thermal degradation behaviour during the test range. The combination of volcanic tuff aggregate and silica-based incorporation of aerogel can be a prospective approach to non-structural lightweight concrete use within the constraints of the current experimental programme, where the minimisation of density and thermal conductivity should be prioritised. The implications related to economics and sustainability are addressed qualitatively.

## Introduction

Energy consumption is an indicator which demonstrates the level of development of a society.^1^. The energy crisis and the hindrance to development have come about due to the rapid increase in population, which has led to the rise in energy demand^2–4^. According to^5,6^, in Turkey, energy consumption in the residences has constituted 24.8 % of the total energy consumption, and 25 % in industry. It is known that buildings are responsible for 35–40 % of total energy consumption.

Lightweight concrete has continued to gain relevance in the contemporary construction sector because of its capability to achieve energy efficiency, structural load reduction and help in sustainable building methodologies^2–4^. Insulation performance is also one of the most important aspects of material development because it directly affects the performance of thermal comfort^7^. One of the weaknesses of traditional concrete is that it is usually not insulating enough due to its strength^8^. In an attempt to address this weakness, studies have participated in the process of integrating industrial by-products and natural resources^9^. The volcanic tuff waste is a porous and silica-containing substance which offers an alternative to the conventional aggregates and is environmental friendly^9–11^. Not only does its use lessen the environmental impact of waste disposal, but it also helps in lightweight properties and enhances thermal resistance of concrete, which is in line with the requirements of global sustainability^12^.

Though silica aerogel is frequently mentioned in the literature due to its very low thermal conductivity and highly porous microstructure, its purpose in cement-based composite depends on its incorporation into the blend^13–16^. The aerogel in the current work was taken in the form of a porous filler of low weight pre-introduced in proportion to mixtures, excluding its use as a nanoscale reactive additive in the cement chemistry^17,18^. Consequently, the study has a restricted scope of assessment of the effects of the chosen levels of incorporation of the aerogel on the unit weight, thermal conductivity, ultrasonic pulse velocity, compressive strength, water absorption, porosity and thermal decomposition behaviour within the adopted experimental matrix^19–21^. There are several research works on energy-efficient building designs and the types of materials used in that regard^22,23^. Nevertheless, further research is required to study the contribution of lightweight concrete types to energy efficiency as well as the improvement of the insulation properties of these concretes^9^. The important aspect of this study is their creativity in bringing nanomaterials such as silica aerogel into lightweight concrete and investigating their impacts on insulation properties thoroughly^24^.

In recent years, applications of high-insulation materials aimed at increasing the thermal insulation value of buildings have increased^25,26^. The low unit weight of concrete enhances its thermal insulation performance^27^. Therefore, materials such as pumice^14,15,28^, volcanic tuff ^29–32^, diatomite^30,31^, polyurethane^14–16^, expanded polystyrene^33–36^ and expanded clay^20,21^ have become popular in the production of lightweight or ultra-lightweight concrete^35^. However, there are some gaps in the literature regarding using these materials. More research is needed, especially on the reuse of waste materials and their effects on the insulation properties of concrete.

The potential application of silica aerogel as an additive in cement-based materials as a thermal insulator has been studied before. Indicatively, other researchers also claimed the incorporation of aerogel results in a major decrease in thermal conductivity and a minor decrease in mechanical strength because aerogel has a highly porous structure^37,38^. Equally, the use of volcanic aggregates and volcanic tuff materials as lightweight aggregates in concrete to enhance density reduction and thermal resistance properties has been investigated in other studies^29,32^. Nevertheless, these materials have been separately studied in the majority of these studies, with experimentation of either the incorporation of aerogel or the application of volcanic aggregates on its own^39^^,^^40^. To better illustrate the distinction between previous studies and the present research, a comparative summary of representative studies is presented in Table 1.

**Table 1:** Comparison of previous studies using aerogel or volcanic aggregates in concrete and the approach of the present study.

**Study type**	**Material used**	**Main objective**	**Key findings**
Aerogel-based concrete studies	Silica aerogel as an additive	Improve thermal insulation of cement composites	Significant reduction in thermal conductivity, but a decrease in compressive strength
Volcanic aggregate concrete studies	Volcanic tuff, scoria, pumice	Produce lightweight concrete with improved thermal resistance	Lower density and improved insulation compared to conventional concrete
Present study	A combination of volcanic tuff waste and synthesised silica aerogel	Investigate the combined effect on insulation performance and structural behaviour	Simultaneous use of waste volcanic tuff and aerogel to improve thermal insulation while utilising sustainable waste resources

Accordingly, the aim of this study is to evaluate the influence of silica aerogel incorporation level, cement dosage, and water-cement ratio on the measured physical and mechanical properties of lightweight concrete produced with Bayburt stone aggregate^41^^,^^42^. The study does not isolate the independent effect of volcanic tuff through a separate comparative aggregate series; instead, it examines the performance of aerogel-containing lightweight concrete prepared with Bayburt stone (BT)as the aggregate system used throughout the experimental program^43^. As such, the current study contrasts the past studies by examining how waste volcanic tuff and synthesised silica aerogel can be used as a composite in lightweight concrete to determine their synergies in relation to insulation properties, density minimisation, and mechanical properties^44^.

### Materials

In this study, Bayburt stone (BT), a white-colored volcanic tuff group from Bayburt province in Turkey’s Black Sea region, was used as the silicon source for silica aerogel synthesis and as a lightweight aggregate material in the preparation of silica aerogel-containing lightweight concrete (SAKHB)^45^. The chemical, physical, and mechanical properties of BT are presented in Table 2^46–48^. It is important to note that BT is an aggregate volcanic material as opposed to a pozzolanic supplementary cementitious material; the values of chemical composition are given to characterise BT.

**Table 2:** Chemical, physical, and mechanical properties of BT.

Physical and mechanical properties				
Oxides	(%)	Feature	Unit	Value
SiO_2_	68.92	Hardness	(Mohs)	4-5
Al_2_O_3_	11.96	Unit Weight	(gr/cm^3^)	1.69
Fe_2_O_3_	0.34	Water Absorption at Atmospheric Pressure	(by weight %)	12.2
SiO_2_+Al_2_O_3_+Fe_2_O_3_	81.22		(by volume %)	20.6
CaO	3.85	Water Absorption at Boiling Water	(by weight %)	13.1
MgO	1.29		(by volume %)	22.0
SO_3_	0.21	Apparent Porosity	(%)	20.6
Na_2_O	0.23	Frost Loss	(%)	0.22
K_2_O	2.38	Bulk Density	(%)	70.1
Total	99.31	Porosity Degree	(%)	29.9
Loss on Ignition	10.13	Average Wear	(cm^3^/50cm^2^)	25.6

In this study, the Bayburt stone (BT) was considered as a lightweight volcanic aggregate and silica source to produce aerogel, but not as a supplemental cementitious material. Consequently, the oxide values are not reported as compliance requirements to the pozzolanic standards and are only provided as an aid to characterise the material. Portland cement of type CEM I 42.5 R (TS EN 197-1)was used in the experiments in study V. G. Parale^48^^,^^49^. The properties of the cement provided by the supplier are presented in Table 3.

**Table 3:** Physical, chemical, and mechanical properties of cement.

Analysis	Oxides	Value	Analysis	Experiments	Value
Chemical (%)	CaO	63.5	Physical	Specific surface area (cm^2^/g)	3320
	Al_2_O_3_	5.35		volume expansion (mm)	1.2
	Fe_2_O_3_	3.30		Water requirement (g)	28.5
	SiO_2_	20.41		Density (g/cm^3^)	3.12
	SO_3_	2.93		Initial setting time (min)	163
	MgO	1.65		Final setting time (min)	240
	Na_2_O	0.15	Mechanical	Day	MPa
	Cl	0.011		2	28.2
	K_2_	0.71		7	42.7
	HCl	0.28		28	51.4

Silica aerogel synthesis utilised hydrochloric acid (HCl) for surface modification, trimethylchlorosilane (TMCS) for surface modification, ethanol (MeOH CH₃OH), and n-heptane (C₇H₁₆) for solvent exchange, and sodium hydroxide beads as the sodium precursor provided by Sigma-Aldrich.

### Silica aerogel synthesis

**Fig. 1:**
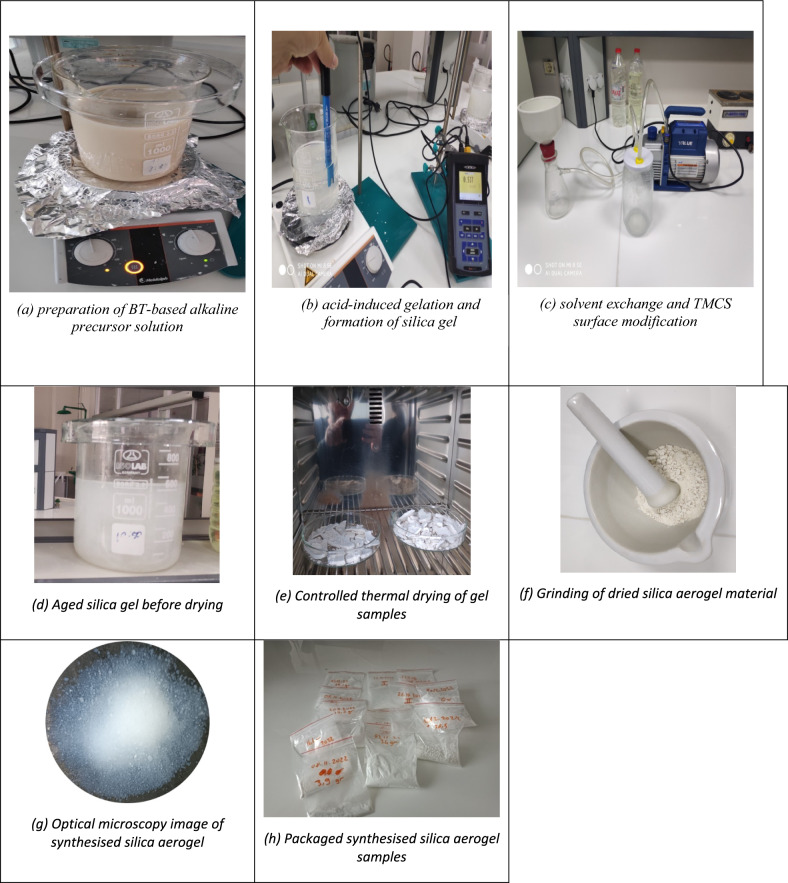
Process diagram for the preparation of aerogels.

Silica aerogel synthesis was carried out using the widely used sol-gel technique^22,23,49–51^. The applied synthesis process is shown in Fig. 1.

The synthesis of the silica-based gel material was achieved using an alkali extraction and sol-gel pathway in which BT powder was used. A batch containing 40g BT powder was combined with distilled water and sodium hydroxide solution in order to dissolve the silica-bearing phase and create a precursor solution with a high content of sodium-silicate. The mixture was heated at 150 °C for a period of 4 h and was filtered under vacuum in order to eliminate dissolved solids^52^. A continuous stirring of the filtrate with 5 M HCl till the pH was found to be about 1 was carried out to induce the formation of the gel. The wet gel was then allowed to gel, followed by a period of two weeks at room temperature (around 21 °C). The gel was put into four washes of distilled water. Solvent exchange subsequently followed in that order ethanol/water, ethanol/TMCS and n-heptane^53,54^. The gel surface was modified by the TMCS treatment in order to minimise the number of surface hydroxyl groups. The drying was done slowly at room temperature, then at 90 C (or 4-8 h) and then at 125 °C (or 24 h). Each batch of synthesis was done at the same temperature, pH, drying, washing and solvent-exchange^55^. Since the current research did not measure BET surface area, bulk density, and pore-size-distribution, the synthesised product can be described in the discussion as a conservatively silica-based aerogel-type material determined by XRD, FT-IR, and microscopy-based observations^56,57^. The exact solid-to-liquid ratio and NaOH solution concentration should be added here if available from the lab record to ensure full reproducibility.

### Experimental design

Experimental design is an approach that can be employed to model and analyse the effects of variables.^58^. The Taguchi method was the method applied in this study in order to identify the effective parameters and levels^59^. The Taguchi method gives an opportunity to evaluate a variety of parameters using fewer experiments and retains the possibility of studying the impact of each variable on the measured results. Initial trials on lightweight concrete specimens established the parameters and levels of Silica Aerogel Containing Lightweight Concrete (SAKHB; 0%-1%-2%-3%), Cement Dosage (C; 300-350 kg/m^3^), and Water-Cement Ratio (W/C; 0.40-0.45), and eight experiments were chosen based on the L8 (4^1^×2^2^) orthogonal array table (Table 4).

**Table 4:** Applied orthogonal array L_8_ (4^1^*2^2^).

Mixture code	Parameters and Levels					
	A	B	C	Aerogel ratio (%)	Cement dosage (kg/m^3^)	Water-Cement ratio (%)
SA030040	1	1	1	SAKHB0	C1	W/C1
SA035045	1	2	2	SAKHB0	C2	W/C2
SA130040	2	1	1	SAKHB1	C1	W/C1
SA135045	2	2	2	SAKHB1	C2	W/C2
SA230045	3	1	2	SAKHB2	C1	W/C2
SA235040	3	2	1	SAKHB2	C2	W/C1
SA330045	4	1	2	SAKHB3	C1	W/C2
SA335040	4	2	1	SAKHB3	C2	W/C1

The chosen aerogel replacement ratios, 0-3%, were calculated according to initial experiment mixtures and previous research studies that reported that even small portions of aerogel may be a factor that can greatly affect thermal insulation because of its porous structure^37,38^. The maximum aerogel percentage was not included in the study since the addition of too much aerogel can greatly decrease the mechanical strength of the concrete and can impair the workability and homogeneity of the mixtures. Thus, the chosen range was deemed suitable in assessing the joint influences of aerogel content, cement dosage, and water-cement ratio on the physical and mechanical characteristics of lightweight concrete^60^.

### Preparation of mixtures and SAKHB samples

The levels of the aerogel incorporation of 0%, 1%, 2% and 3% define the desired levels of the mixture design applied in the Taguchi matrix implemented by changing the aggregate-phase quantities to keep the total mixture volume constant (1m^3^ ). Table 5 illustrates that there were proportional decreases in the quantities of the Bayburt stone aggregate as a result of increasing the content of aerogel. Thus, the aerogel was not a replacement for cement but a lightweight porous filler to be added at the level of mixture design. Table 5 shows the mass quantities of each mixture used in the experiment to allow reproducibility of the tested formulations.

**Table 5:** Required material quantities (kg) for 1 m^3^ mixture.

**Mixture code**				Aggregate (Bayburt Stone)		
	Aerogel	Cement dosage	Water-cement ratiooranı	(0-4) mm Fine	(4-8) mm Coarse	(8-11.2) mm Coarse
SA030040	0	300	0.40	940.8	156.8	548.8
SA035045	0	350	0.45	876.6	146.1	511.3
SA130040	7.25	300	0.40	931.4	156.8	548.8
SA135045	6.75	350	0.45	867.8	146.1	511.3
SA230045	14.08	300	0.45	904.3	153.8	538.3
SA235040	13.70	350	0.40	879.6	149.6	523.6
SA330045	20.92	300	0.45	895.1	153.8	538.3
SA335040	20.35	350	0.40	870.6	149.6	523.6

**Fig. 2:**
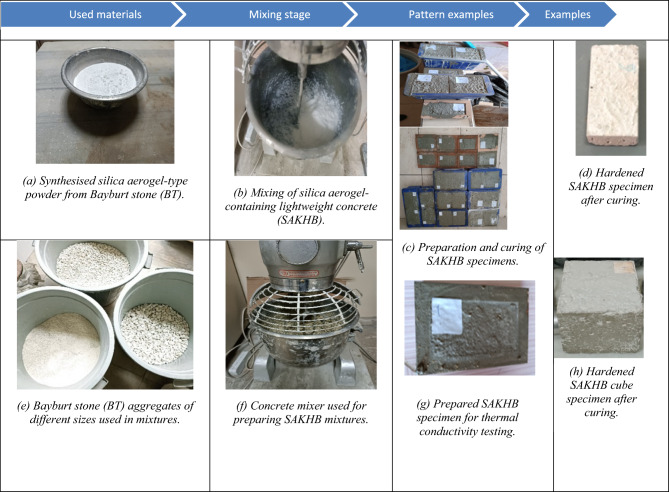
Sample preparation process. Optical microscopy images of SAKHB samples: (**a**) reference mixture without silica aerogel, (**b**) mixture containing 1% silica aerogel, (**c**) mixture containing 2% silica aerogel, and (**d**) mixture containing 3% silica aerogel. The images show the pore structure, distribution of silica aerogel particles, and the interface between the aerogel-containing regions and the cement matrix.

To achieve the same, BT aggregates were soaked for 30 minutes before mixing^61^. Figure 2 shows the sample preparation processes. The SAKHB mixtures were prepared by substituting fine aggregates, coarse aggregates, cement and silica aerogel homogeneously in sequence^62^. The mixture was then mixed with water. According to the requirements of the test devices, the prepared mixtures were placed in 100x100x100 mm cube moulds and 120x60x30 moulds^63^. After the ASTM C511-03 standard, the mixtures were cured in the lime-saturated water for 28 days at (20 ± 1 °C)^64^.

The special consideration during the mixing process was to maintain the homogeneity of the mixtures and to have uniform dispersion of silica aerogel particles in the concrete matrix. Silica aerogel is very porous and lightweight, which means that its excessive use can affect the workability and slump of fresh concrete mixtures. Thus, the aerogel was added slowly in the mixing process so that the particles could not be agglomerated, and they could also not be segregated. Arrangement of mixtures and controlled pouring of materials were useful in the preservation of sufficient workability and homogenization of aerogel particles in mixtures. A discussion of the slump behaviour and its effect on the performance of the mixture is presented in the results section.

The silica aerogel introduced in this paper was a lightweight porous solid that was put into the concrete by the adjustment of mixture proportions, but not as a chemical nano-additive that altered the cement hydration at trace levels. The aerogel was treated like a particulate filler in the aggregate phase of the mixture design, and the aggregate amounts were varied to sustain the 1m^3^ target mixture yield.

### Characterization methods

The synthesized silica aerogel and produced several analyses characterized by SAKHB samples. The structural characterization of the synthesized silica aerogel was carried out using XRD, FT-IR, TEM microscopy, optical microscopy and real density determination. XRD analysis was done on Bruker D8 Discover model device within the 2 theta 5°-95° range to find crystal structure, particle size, and average value of the material. However, a silica aerogel prepared in this manner was examined by FT-IR analysis using a Perkin Elmer Spectrum Two model IR device with a Diamond ATR kit, which used a solid sample obtained from the synthesized silica aerogel. Real-density determination was carried out using a Micromeritics Accupyc II 1340 Gas Pycnometer, and the morphological properties of the nanoparticles were evaluated using a FEI Talos F200S HR-TEM (200 kV) system. The SAKHB samples were optically examined using an optical microscope with an NMM-800/820 series. The material’s weight change and heat flow under a temperature condition were analyzed using thermal gravimetric analysis (TGA) using a Perkin Elmer Diamond system.

Characterisation programme used in this paper was XRD, FT-IR, TEM, optical microscopy, and density measurements with a gas pycnometer. They are enough to reveal silica-related chemistry and that particular morphology of fine porous particulate, but will not determine a complete aerogel classification in the strict textural sense since the bulk density, BET surface area, pore-size distribution, and direct porosity measures of the synthesised powder were not measured. Thus, the content is construed in a conservative way in this manuscript as a silica-based aerogel-type material that is prepared with the help of a sol-gel pathway.

### Test methods

Tests were conducted on 100x100x100 mm specimens to measure density, water absorption, and porosity (TS EN 12390-7), ultrasonic pulse velocity (ASTM C 597) and compressive strength (TS EN 772-1)^65–68^. Thermal conductivity tests were conducted on 120x60x30 mm specimens (ASTM C1113-90). Thermal conductivity measurement was performed using the KEM QTM-500 model Thermal Conductivity Meter. The specimens were left to equilibrate in laboratory conditions before thermal conductivity measurements, in order to minimise the effect of free water on the property of heat transfer. The measurements were done at room temperature under controlled laboratory conditions. The tests using the Matest C372M device were performed using ultrasonic pulse velocity. Later, an NMM-800/820 series optical microscope was used to examine some specimens by optical microscopy. It has been known that the moisture content can affect the thermal conductivity values because the thermal conductivity of water is greater than that of air in the pore structure of concrete. Thus, the same level of moisture in all of the tested specimens contributed to the comparability and validity of the obtained thermal conductivity measurements.

## Results and discussion

In two stages, this study was designed. The sol-gel method was used to synthesise a silica aerogel out of Bayburt stone waste, and the structural and morphological properties of the synthesised material have been characterised. Afterwards, lightweight concrete (SAKHB) containing synthesised silica aerogel was made in the second stage, and the samples’ structural, morphological, mechanical and physical properties were examined^69^. Below are the data obtained.

### Silica aerogel analysis results

#### XRD analysis

**Fig. 3:**
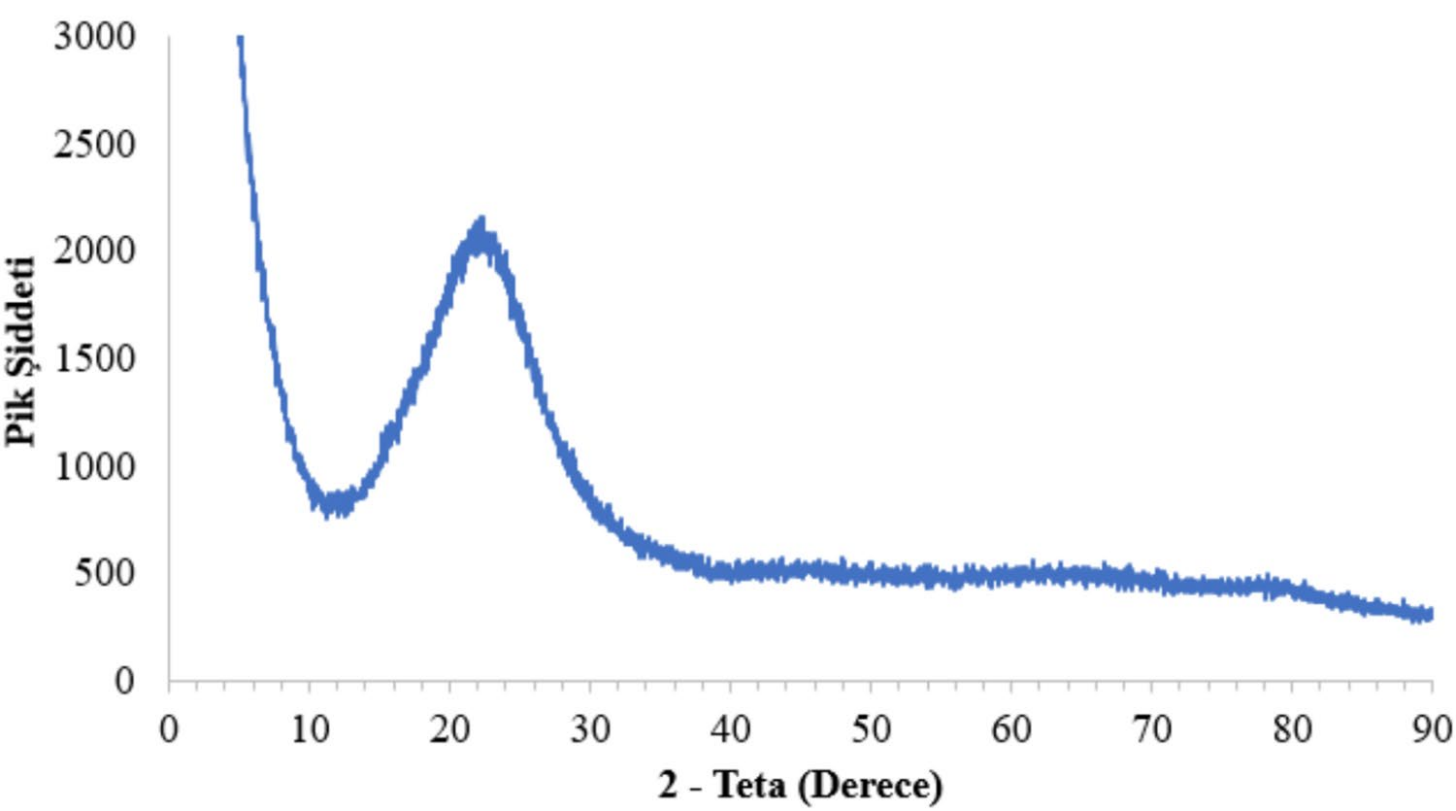
XRD graph of the synthesised silica aerogel.

X-ray diffraction (XRD) analysis was conducted to determine the crystalline structure of the synthesised silica aerogel-type material. The analysis was performed using a Bruker D8 Discover diffractometer with Cu-Kα radiation (λ = 1.5406 Å) as shown in Fig. 3. The measurements were carried out over a 2θ range of 5°–95° with a step size of 0.02° and a scanning rate of 2°/min. The samples were prepared in powder form and placed on a flat sample holder prior to analysis. The obtained diffraction patterns were used to identify the amorphous silica structure of the synthesised material^70^.

A peak was formed at 21.5°, which is the same characteristic peak of amorphous silica aerogel (2θ from 20° to 30°)^71^. Another noteworthy feature in the graph is the sufficiency of the washing process. NaCl peaks were decreased by the number of washings in Ciza’s study.^72^. This says that enough distilled water was utilised during synthesis. In their XRD analyses of silica aerogel syntheses found in the literature, Zhou et al. found a peak at 23.2°^73^, Wu et al. at 22°^74^, Rahmani et al. at 20°^75^, and Saraç between 22° and 23°^76^. XRD sample preparation conditions, impurities in the sample and deviations from the X-ray tube can cause ±1-2° deviations in peaks. In XRD analysis, such deviations are also considered normal. It follows from the analysis that silica aerogel is successfully made.

#### TEM analysis

**Fig. 4:**
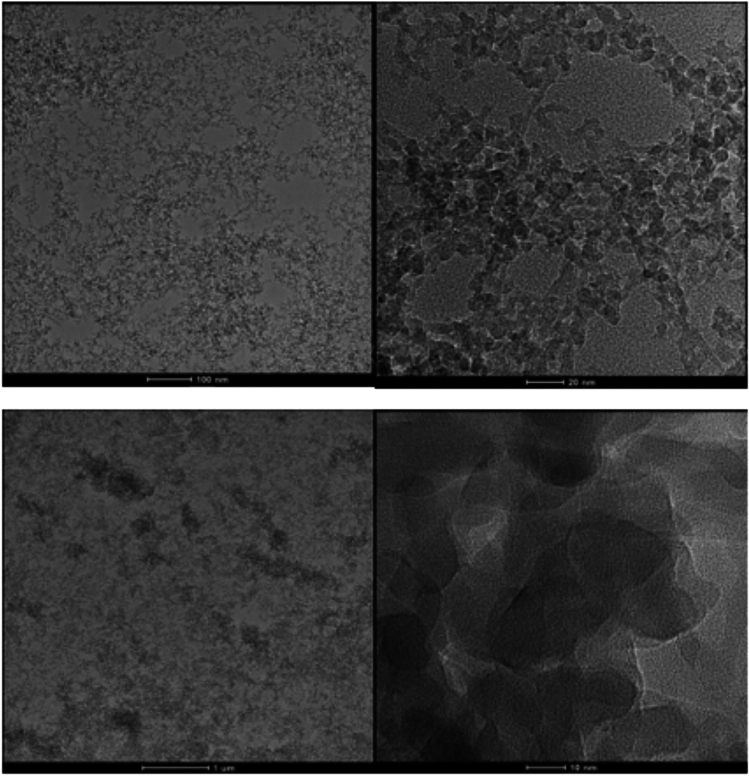
TEM images of the synthesised silica aerogels.

TEM analysis is used to determine the morphological properties of the material. The TEM images of the synthesised silica aerogel are shown in Fig. 4.

Figure 4 shows TEM images of the synthesised silica aerogels, demonstrating approximately 10 nm size, homogeneous shape, and particle size distribution, similar to other similar works.^77–79^. Another result of these images is that there is no sintering or agglomeration of particles and no interactions between particles. Due to these features, the synthesised silica aerogel particles are supposed to feature a structure that can easily disperse^80^.

#### FT-IR Analysis

**Fig. 5:**
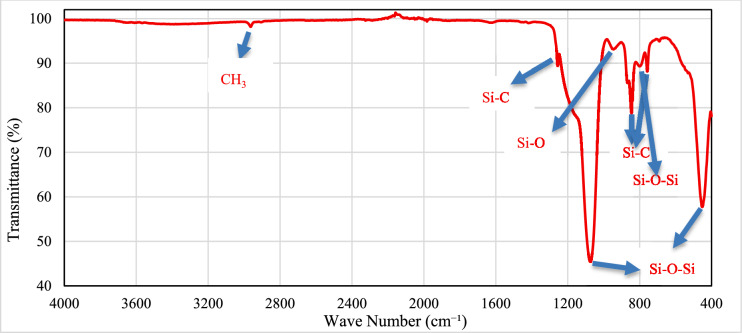
FT-IR spectra of the synthesised silica aerogel.

FT-IR spectroscopy was applied to characterise the composition and interactions of the synthesised silica aerogel, and the results are shown in Fig. 5. FT-IR spectrum data indicate that the main characteristic data of silica aerogel is the Si-O-Si vibration type, occurring between 1075 cm^−1^ and (1250-1000 cm^−1^)^81^. Additionally, other spectrum peaks represent CH3 (2963 cm^−1^), Si-C (1255, 845, and 757 cm^−1^), and Si-O (946 cm^−1^) vibrations, indicating successful synthesis of silica aerogel consistent with the literature.^82–87^.

#### Density and optical microscopy examinations

The pycnometer weight of the material synthesised was determined to be 1.8774 +/-0.0242 g/cm 3. Since the gas pycnometer values are a measure of skeletal or true density and not bulk density, this finding should not be viewed as a direct indication of ultralow-density aerogel structure^88^. Rather, it is a measure of the solid framework density available to the measuring method. The present study did not find the bulk density and BET-based parameters of the porosity; the density finding is presented here as skeletal-density information only and is discussed in combination with the XRD, FT-IR, and microscopy data^89^.

### SAKHB test results

The density, ultrasonic pulse velocity, thermal conductivity, and compressive strength values of the lightweight concrete samples containing silica aerogel are presented in Table 6.

**Table 6:** Properties of lightweight concrete samples containing silica aerogel.

Mixture code	Unit volume weight values (kg/m^3^)		Thermal conductivity values (W/mK)		Ultrasonic pulse velocity values (km/s)		Compressive strength values (MPa)	
	Results	Average	Results	Average	Results	Average	Results	Average
SA030040	1722	1723	0.6921	0.7131	3.36	3.26	15.82	15.04
	1735		0.7241		3.11		14.85	
	1712		0.7231		3.32		14.45	
SA035045	1730	1705	0.7235	0.7272	3.22	3.15	16.55	16.35
	1690		0.7315		3.00		15.85	
	1695		0.7266		3.23		16.66	
SA130040	1641	1642	0.7010	0.6846	3.05	3.11	12.80	13.64
	1660		0.6790		3.23		14.01	
	1625		0.6738		3.06		14.11	
SA135045	1607	1604	0.6031	0.624	2.87	2.98	14.90	14.22
	1620		0.6327		3.03		14.05	
	1585		0.6362		3.05		13.71	
SA230045	1516	1507	0.5136	0.4919	2.28	2.38	12.07	11.96
	1525		0.4890		2.42		12.03	
	1480		0.4731		2.46		11.52	
SA235040	1512	1523	0.4430	0.4252	2.49	2.41	11.35	11.06
	1517		0.4125		2.36		10.97	
	1540		0.4201		2.38		10.87	
SA330045	1433	1405	0.4710	0.4628	2.29	2.32	9.85	9.19
	1390		0.4540		2.38		8.72	
	1392		0.4634		2.30		9.01	
SA335040	1370	1380	0.4105	0.3908	1.98	2.02	8.91	9.72
	1365		0.4039		2.15		10.30	
	1405		0.3580		1.93		9.95	

#### Unit volume weight values

**Fig. 6:**
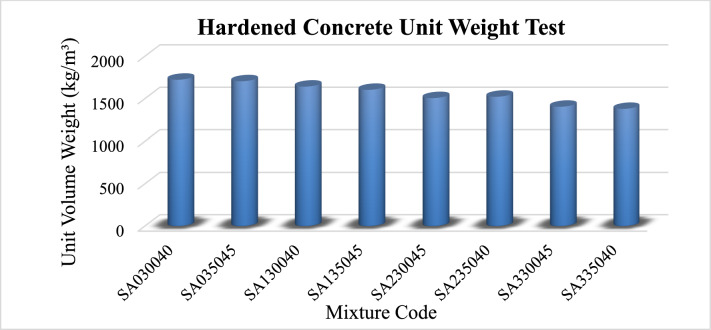
Unit weight test results of hardened concrete in SAKHB.

Aerogel is a low-density solid material. Increasing the proportion of silica aerogel in the mixture reduces the unit weight of fresh and hardened concrete. The density of the hardened concrete decreases from 1723 kg/m^3^ to 1380 kg/m^3^ with the increase in silica aerogel replacement, as shown in Table 6 and Fig. 6. These results indicate that the produced samples meet the standards for lightweight concrete.

#### Thermal conductivity test results

**Fig. 7:**
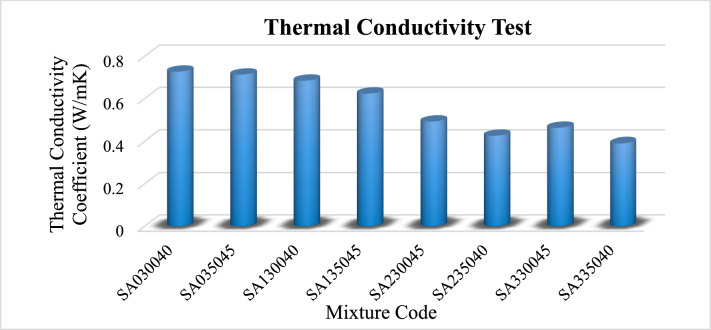
Thermal conductivity test results in SAKHB.

The responses measured are internally consistent among the tested mixtures. Unit weight reduced with aerogel incorporation had to 1380 kg/m^3^ with 1723 kg/m^3^, thermal conductivity went down to 0.3908 W/mK with 0.7272 W/mK, and ultrasonic pulse velocity and compressive strength declined. This trend indicates that the inner porous structure corresponding to a higher concentration or density of aerogels hinders the transfer of heat, but on the other hand, compromises the continuity and rigidity of the concrete matrix. Thus, there is a mechanical penalty associated with the thermal benefit, and this ought to be taken into account in delimiting the range of applications of these mixtures Fig. 7.

The decrease in thermal conductivity can also be viewed in the context of other physical characteristics of the produced mixtures. The higher the percentage of silica aerogel, the lower the density of the concrete because silica aerogel is of extremely low density and possesses a porous structure. This decrease in density is accompanied by the growing porosity of the overall concrete matrix, as presented in the results of the porosity test. These extra air voids and nanoporous framework of silica aerogel lower the level of heat transmission through the material since the air-filled pores possess extremely low thermal conductivity in contrast to the solid matrix. Thus, the overall impact of reduced density, enhanced porosity, and low thermal conductivity inherent to silica aerogel is the source of the enhanced performance of insulation observed in the SAKHB samples.

#### Ultrasonic pulse velocity test results

**Fig. 8:**
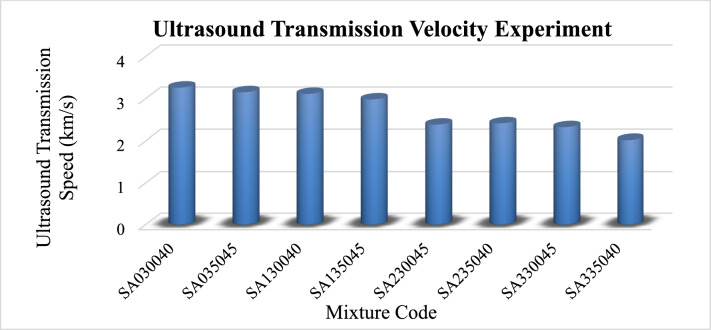
Ultrasonic pulse velocity test results in SAKHB.

Table 6 presents the ultrasonic pulse velocity values of the produced samples, and Fig. 8. Ultrasonic pulse velocity is related to density. As the porosity of the sample increases, ultrasonic pulse velocity decreases. As the amount of aerogel in the sample increases, the porosity of the aerogel increases along with porosity. The results in the figure confirm this. Values like void ratio, compressive strength and water absorption of the sample can be interpreted through ultrasonic pulse tests.

#### Compressive strength test results

**Fig. 9:**
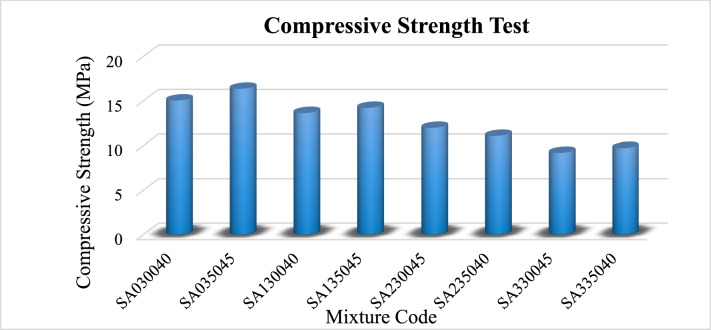
Compressive strength test results in SAKHB.

The values in Table 6 and Fig. 9 show that compressive strength ranges from approximately 16 MPa to 9 MPa. As the amount of silica aerogel increases, there is a decrease in compressive strength in SAKHB samples, indicating a negative impact of silica aerogel on the binding property of cement. The brittle nature, weak bonding effect, and low strength of silica aerogel can affect the mechanical strength of concrete mixtures^90^. The results support the literature data on the impact of aerogel on compressive strength.^8,34,37,38,43,91,92^

The compressive strength of silica aerogel is reduced by its incorporation, but the enhanced thermal insulation properties of this material could offer some benefits in some construction projects where the main criterion is not the structural strength of the material. Silica aerogel-enriched lightweight concrete can be used in this situation in non-load-bearing structural elements, insulation panels, facade insulation systems, and lightweight partition blocks. The performance of the thermal insulation may offset the decrease in mechanical strength in these applications and can also be used to promote energy performance in the buildings.

#### Water absorption (by weight) and porosity test results

**Fig. 10:**
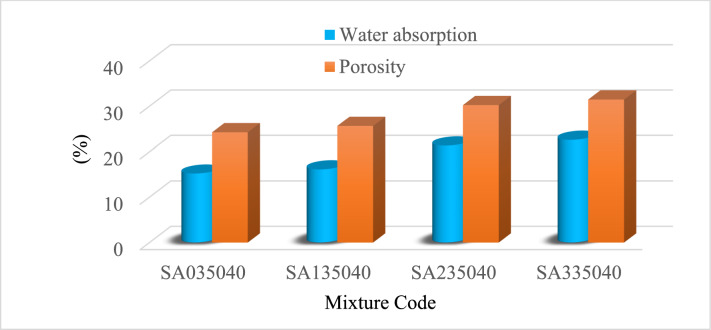
Water absorption and porosity test results in SAKHB.

The permeability of a concrete structure has a significant impact on its strength. The porous structure of lightweight concrete can increase water absorption levels. ANOVA analysis determined the optimal levels of parameters, and water absorption (by weight) and porosity tests were conducted on SAKHB samples according to these levels. The results are presented in Table 7 and Fig. 10.

**Table 7:** Water absorption (by weight) values in SAKHB.

Mixture code	Water absorption values (%)	Porosity values (%)
	Average	
SA035040	15.21	24.24
SA135040	16.14	25.65
SA235040	21.33	30.25
SA335040	22.59	31.48

As the proportion of silica aerogel increases in lightweight concrete samples, the water absorption and porosity ratios increase^83^. This observation probably happens when air bubbles are found around the silica aerogel^93^. However, it is thought that the porosity and absorption values of the concrete could be increased by the air bubbles forming around the silica aerogel in the concrete^91,94,95^. Furthermore, the porosity ratio also increases in plaster and foam concrete with silica aerogel^72,96^. The physical modelling of the reaction of silica aerogel in concrete and air bubbles around it^97^^,^^98^.

The growth in the porosity and water absorption could also have an effect on the thermal characteristics within the concrete mixtures. Increased porosity is more likely to add more air-filled pores in the concrete mass, which translates to the reduction of thermal conductivity and enhancement of insulation behaviour. Nevertheless, these pores can be filled with moisture, and therefore, the thermal conductivity of the material can also increase since water is more thermally conductive than air. Thus, porosity, moisture content and aerogel incorporation have a significant effect on the overall thermal insulation performance of the SAKHB samples.

#### Optical microscopy examinations

Figure 2 shows representative optical microscopy images of SAKHB samples labelled (a)–(d). The reference sample appears denser with fewer voids, while aerogel-containing samples show more pores around aerogel particles^84^. The images confirm aerogel distribution in the cement matrix and support the observed increase in porosity and reduced density and thermal conductivity. To observe their general structures, SAKHB samples were examined under an optical microscope at 50X, 100X, and 200X magnifications. Macrostructure and optical microscope images of SAKHB samples indicate that the reference sample has certain voids, and adding silica aerogel provides voids around the aerogel particles. It is also observed that silica aerogel tends to concentrate in certain concrete parts. This is prevented by using a homogeneous mixture, as the increased concentration in a particular region alone can result in higher porosity.

The distribution of silica aerogel particles in the concrete matrix is demonstrated by representative optical micrographs of the micrographs obtained at various magnifications, which are labelled accordingly. The micrographs indicate that the aerogel particles are typically enclosed within tiny vacuities owing to the very porous nature, which helps in the development of further air-filled pores in the concrete. These photos also assist in the physical verification of the aero-gel particle scattered dispersion and interplay with the cement matrix around it. The label micrographs also help to determine the area of aerogel as well as the pore structure surrounding it, which gives a clearer picture of what microstructural changes are brought about by incorporating aerogel.

#### Thermogravimetric Analysis (TGA)

**Fig. 11:**
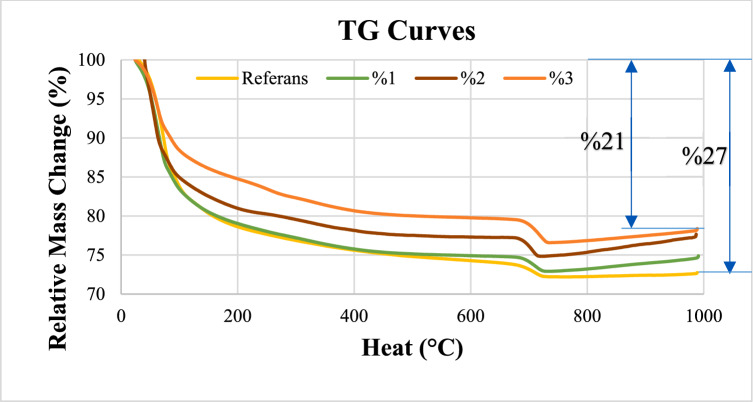
TG graph of SAKHB samples.

Thermogravimetric (TG) analysis of SAKHB samples was carried out using a Perkin Elmer Diamond thermal analysis system. The samples were heated from 25 °C to 1000 °C at a heating rate of 10 °C/min under an air atmosphere. The TG curves represent the change in mass (%) of the samples as a function of temperature (°C), and the results are presented in Fig. 11.

Figure 11 shows that the total mass loss decreases as the silica aerogel content increases from 0% to 3% in SAKHB samples. The observed mass loss in the TG curves can be attributed to different thermal decomposition stages of cement-based materials. The initial mass loss at lower temperatures is mainly associated with the evaporation of physically bound water. The intermediate mass loss corresponds to the dehydration of hydration products such as calcium hydroxide and other cementitious phases. At higher temperatures, further mass changes may occur due to the decomposition of remaining mineral components.

The reduction in total mass loss with increasing aerogel content indicates differences in thermal decomposition behaviour among the mixtures. This may be related to the presence of thermally stable silica structures and changes in pore structure introduced by the aerogel. However, this result should not be directly interpreted as evidence of improved long-term durability. A more detailed stage-wise TG analysis and additional durability tests would be required to establish a direct relationship between thermal behaviour and durability performance.

#### Optimisation results and ANOVA analysis

To optimise the density, ultrasonic pulse velocity, thermal conductivity, and compressive strength values of the lightweight concrete samples containing silica aerogel, Minitab 19 software was used to select separate parameters for each experiment, and analyses were conducted. The signal-to-noise (S/N) ratios obtained after the analysis are presented in Table 8. S/N ratios obtain the “optimum” condition to save time and reduce costs^99^.

**Table 8:** S/N ratios of SAKHB test results.

Mixture code	Slump values	Unit weight (S/N Ratio)	Ultrasonic pulse velocity (S/N Ratio)	Thermal conductivity (S/N Ratio)	Compressive strength (S/N Ratio)
SA030040	20.0000	-64.7257	70.2777	2.9370	24.2730
SA035045	20.9065	-64.6345	69.9662	2.7669	23.5450
SA130040	20.2567	-64.3075	69.8692	3.2913	22.6963
SA135045	21.0616	-64.1041	69.4989	4.0963	23.0580
SA230045	20.4238	-63.5623	67.5607	6.1625	20.8790
SA235040	20.9844	-63.6540	67.6475	7.4281	21.3377
SA330045	20.6685	-62.9535	67.3285	6.6921	19.2710
SA335040	21.1381	-62.7976	66.1156	8.1609	19.7533

Taguchi analysis was conducted on the orthogonal array of L8 based on the values of the measured average responses of each mixture. No replicated orthogonal arrays were done. Thus, the error term of the ANOVA is an unexplained residual variation in the fitted model of the selected factors and levels that is to be interpreted with caution due to the limited scale of the design. The Taguchi ANOVA analysis, used in this paper, is not aimed at making a claim of a fully resolved interaction predictive model but to determine the approximate factor effect in the tested design space.

After determining the S/N ratios using the Taguchi method, ANOVA analysis was conducted to find the effect ratio of the parameters in each experiment on the SAKHB samples, and the results are presented in Table 8.

The R^2^ and R^2^ (adj) values for all experiments in Table 9 indicate a perfect correlation between actual and corrected values, as they are close to 1

**Table 9:** ANOVA analysis results of the SAKHB test results.

	Source	DF	SS	Effect	MS	F-value	P-value
Hardened concrete unit weight	Aerogel ratio (%)	3	115522	98.87%	38507.5	148.03	0.007
	Cement dosage (kg/m^3^)	1	528	0.45%	528.1	2.03	0.290
	Water-cement ratio (%)	1	276	0.24%	276.1	1.06	0.411
	Error	2	520	0.45%	260.1		
	Total	7	116847	100.00%			
	R^2^ / R^2^ (adj)	99.55% / 98.44%					
Ultrasonic pulse velocity	Aerogel ratio (%)	3	1494036	96.06%	498012	37.18	0.026
	Cement dosage (kg/m^3^)	1	34322	2.21%	34322	2.56	0.251
	Water-cement ratio (%)	1	144	0.01%	144	0.01	0.927
	Error	2	26788	1.72%	13394		
	Total	7	1555291	100.00%			
	R^2^ / R^2^ (adj)	98.28% / 93.97%					
Thermal conductivity	Aerogel ratio (%)	3	0.124954	94.87%	0.041651	59.41	0.017
	Cement dosage (kg/m^3^)	1	0.004287	3.26%	0.004287	6.12	0.132
	Water-cement ratio (%)	1	0.001063	0.81%	0.001063	1.52	0.343
	Error	2	0.001402	1.06%	0.000701		
	Total	7	0.131706	100.00%			
	R^2^ / R^2^ (adj)	98.94% / 96.27%					
Compressive strength	Aerogel ratio (%)	3	45.5266	97.12%	15.1755	33.75	0.029
	Cement dosage (kg/m^3^)	1	0.019	0.04%	0.0190	0.04	0.856
	Water-cement ratio (%)	1	0.4325	0.92%	0.4325	0.96	0.430
	Error	2	0.8992	1.92%	0.4496		
	Total	7	46.8772	100.00%			
	R^2^ / R^2^ (adj)	98.08% / 93.29%					

The error term in the Taguchi-based ANOVA analysis is the variation that could not be attributed to the selected control parameters. Since the L8 orthogonal design is associated with a small number of experimental runs and no further replication was carried out, the error term indicates the remaining variation in the dataset. This limitation does not discount the fact that the results of the ANOVA are useful in terms of informing about the relative significance of the parameters under investigation.

In the case of thermal conductivity, the Taguchi signal-to-noise analysis revealed an optimal combination of 3% aerogel, 350 kg/m^3^ cement dosage, and 0.40 water/ cement ratio. This optimum is a statistically predicted mixture of the factor level design space and was later verified by a validation experiment. In the cases of other responses, the debate below separates the most accurately measured among the experimental combinations and the forecasted optimum combination, which was obtained through Taguchi analysis. According to the ANOVA outcomes, the ratio of aerogel had the greatest impact on the hardened concrete unit weight, ultrasonic pulse velocity, thermal conductivity, and compressive strength, with a contribution ratio of 98.87%, 96.06%, 94.87% and 97.12%, respectively. The highest average ultrasonic pulse velocity of the measured mixtures corresponded to the reference mixture SA030040, and the lowest thermal conductivity corresponded to SA335040. The highest compressive strength was noted in the 0 per cent aerogel mixtures as the aerogel content tended to increase in density, ultrasonic pulse velocity and compressive strength, and decrease thermal insulation performance. Table 10 shows the regression equations used to estimate the hardened concrete unit weight, ultrasonic pulse velocity, thermal conductivity and compressive strength.

**Table 10:** Regression equations in SAKHB.

Equation No	Regression equations
1	Hardened concrete unit weigh (kg/m^3^) = 1928 – 107.25X_1_ – 0.325X_2_ - 235X_3_
2	Ultrasonic pulse velocit (m/s) = 4050 – 375.2X_1_- 2.62 X_2_ + 170X_3_
3	Thermal conductivity (W/mK) = 0.831 – 0.1076X_1_ – 0.000926X_2_ + 0.461X_3_
4	Compressive strength (MPa) = 19.12 – 2.129X_1_ + 0.00195X_2_ – 9.30X_3_

In the regression equations in Table 10, X1, X2, and X3 in the regression equations represent the coded values of the aerogel ratio, cement dosage and water-cement ratio, respectively. The coding is based on the factor levels applied in the L8 design: X1 = aerogel level (0%, 1%, 2%, 3%), X2 = cement dosage level (300 and 350 kg/m 3 ), and X3 = water-cement ratio level (0.40 and 0.45). They are valid only in the factor ranges that are being investigated. The predictions beyond such ranges must be taken with care. According to the S/N data, thermal conductivity values are optimised with 3% aerogel, 350 kg/m^3^ cement, and a 0.40 water/cement ratio. Validation experiments conducted with these mixture ratios are presented in Table 10, consistent with the results in Table 6. Since the design is small, the regression equations are given as empirical screening equations of the ranges studied, and their sufficiency should be viewed in conjunction with the validation experiment instead of being seen as general predictive equations outside the design space studied.

It is important to note that the best combination of parameters found using the Taguchi method reflects a statistically predicted optimum situation using the S/N analysis and not a combination of mixtures actually presented in the original orthogonal arrayas show in Table 11. Thus, further validation experiments were conducted to verify the predicted ideal performance of the mixture. The comparison between predicted and validation results indicates that the Taguchi-based regression models provide reasonable prediction accuracy within the tested parameter ranges, supporting the reliability of the optimisation analysis for the investigated mixture design.

**Table 11:** Validation test results in SAKHB.

	Unit volume weight	Ultrasonic pulse velocity	Thermal conductivity	Compressive strength
Validation test result	1397 kg/m^3^	2150 m/s	0.4012 W/mK	9.9 MPa

#### Cost analysis

The cost analysis of SAKHB in the current paper is qualitative, and it is a partial appraisal of the costs, as opposed to a complete economic analysis. The mixture system comprises Bayburt stone (BT), as a waste-based aggregate source, and silica aerogel, as a lightweight insulating material. Based on the proportions of mixtures utilised in this research, the silica aerogel-containing mixtures have an approximate incorporation of 20 kg/m 3 silica aerogel as the highest percentage incumbent, with the aggregate phase predominantly consisting of BT-based material.

In reality, the economic impacts of SAKHB are primarily based on sourcing costs, processing costs, transportation costs, and the cost of synthesis. BT can offer a green benefit since it is utilised as a waste-based product; nevertheless, economic value is also still conditional on the collection, transportation, and processing needs. In comparison, silica aerogel will likely come at the highest cost sensitivity due to its production process and related treatment steps^85,100^. As such, the cost of direct production of SAKHB mixtures should be more expensive than conventional mixtures of lightweight concrete.

Nevertheless, the current paper lacks an in-depth calculation of cost-per-m 3, a unit price table, a system border, or a sensitivity analysis. This is the reason why no concrete statement is drawn at this point in the question of overall economic gain, lower maintenance cost, lower operational cost, or long-term financial gain. Rather, it is merely indicated in its findings that the increased thermal insulation efficiency manifested in the aerogel-comprising mixtures could potentially have a practical use in areas where minimised thermal conductivity is of more importance^101–103^. A more vigorous economic conclusion would have to be a different form of quantitative techno-economic analysis on the basis of material prices, processing costs, and transportation distance, as well as building-use cases.

## Conclusion and recommendations

This research measured the impact of the addition of silica aerogel on the measured performance of lightweight concrete made using Bayburt stone aggregate. In the mixtures studied, a higher aerogel content decreased hardened density (1723 kg/m 3 in the reference mixture) to 1380 kg/m 3 in the highest-aerogel mixture and thermal conductivity (0.7272 W/mK) to 0.3908 W/mK, a reduction of about 46% compared with the reference mixture. These findings present better thermal insulation performance. Meanwhile, the porosity went up to 31.48%, and compressive strength went down, clearly indicating a trade-off between the insulation advantage and mechanical performance. The TG analysis indicated that the overall mass loss was reduced in mixtures containing aerogels under the thermal programme applied; this is, however, interpreted as a variation in thermal decomposition behaviour and not necessarily a direct indication of long-term durability. These results offer various advantages in practical applications: Silica aerogel-containing lightweight concrete can be used in energy-efficient buildings, making it ideal for green buildings and passive houses. It provides energy-saving and cost-reduction potential, reducing buildings’ heating and cooling costs. Reusing waste materials contributes to environmental sustainability and helps conserve natural resources. It provides a competitive advantage in the market with high insulation performance and low energy consumption. It reduces maintenance and operational costs due to high durability and long lifespan.

### Recommendations

The combination of the measured density, thermal conductivity and strength values provides evidence that the studied mixtures are better suited to non-structural lightweight and insulating applications than to the load-bearing structural use. Future activities ought to involve bulk-density and pore-structure characterisation of the synthesised aerogel-type material, quantitative cost analysis, and specific durability testing to ensure that the limits of its application can be delimited more strictly.

## Data Availability

All data generated or analysed during this study are included in this published article
